# Estimating the number and growth of tobacconists and vape stores in Queensland in the absence of a retailer licensing database

**DOI:** 10.1111/dar.14038

**Published:** 2025-03-03

**Authors:** Hollie Bendotti, David Ireland, Coral Gartner, Henry M. Marshall, Sheleigh Lawler

**Affiliations:** ^1^ School of Public Health The University of Queensland Brisbane Australia; ^2^ Thoracic Research Centre The University of Queensland Brisbane Australia; ^3^ The Australian e‐Health Research Centre Commonwealth Scientific and Industrial Research Organisation Brisbane Australia; ^4^ NHMRC Centre of Research Excellence on Achieving the Tobacco Endgame The University of Queensland Brisbane Australia; ^5^ Department of Thoracic Medicine The Prince Charles Hospital Brisbane Australia

**Keywords:** e‐cigarettes, licensing, retail environment, tobacco, tobacco control

## Abstract

**Introduction:**

In 2022, Queensland had not implemented a tobacco retailer licensing scheme. This study aimed to develop a prototype system to periodically collect geolocations of tobacconists and/or vape retailers (TVR) in Queensland over a 12‐month period.

**Methods:**

The ‘Text Search’ function on Google Maps application programming interface was used to return business information based on a string query with a specific latitude and longitude coordinate (search points) (*n* = 3481). Monthly searches of TVRs were repeated from July 2022 to June 2023. Retailers that likely sold tobacco products but were not specialty stores (e.g., supermarkets) were excluded. Two team members independently and manually checked, confirmed and categorised ‘New’ entries.

**Results:**

From July 2022 to June 2023 confirmed TVRs in Queensland increased by 14.3% from 624 to 713, a mean of 7.4 new TVRs per month. Of the total stores collected in the initial search (July 2022), 71.5% were manually confirmed as TVRs. Most stores were categorised as ‘tobacco only’, yet the proportion of confirmed TVRs categorised as ‘tobacco and vape’ and ‘vape only’ increased and decreased, respectively, over 12‐months.

**Discussion and Conclusions:**

The prototype system effectively identified and tracked the longitudinal growth of tobacco/vape specialty stores in Queensland over 12 months. Future research using the system will analyse TVR density and proximity relative to population characteristics and locations of interest. The system will also provide baseline data to assist compliance following the Queensland smoking product licensing scheme and longitudinal data to supplement evaluations of the state and federal supply policies.

## 1 INTRODUCTION

International evidence highlights the association between tobacco retailer density and geographic location to tobacco product use [1]. Restricting retail availability of tobacco products (supply reduction) is recommended as a tobacco endgame strategy to reduce smoking prevalence and promote health equity [2]. To date, Australia's successful tobacco control measures have largely focussed on reducing the demand for tobacco products rather than the supply [3]. Tobacco/vape retailer licensing schemes have been enacted by most Australian state and territory governments, but there is heterogeneity in their design and fee structures [4]. Retailer licensing schemes have had majority public support since 2004 [4] because they allow government and public oversight of retailer locations, and regulation of products and advertising. These schemes are particularly important for monitoring specialty tobacconists and vape retailers (TVR) which operate independently or as part of a franchise, and whose core business is tobacco product sales. Therefore, there is greater potential for exposure to tobacco/vape products [5, 6], price competition [7], tobacco industry influence, and compliance issues with supply and advertising laws [8, 9]. Moreover, in 2022–2023, 32.2% of Australians (14+ years) who currently smoke obtained tobacco products from specialty retailers, second only to major supermarket chains (36%), and specialty retailers were the most common outlet among young people (15–24 years: 31.9%; 25–29 years 32.5%) [10]. Licensing scheme geographic data (e.g., street address) allows spatial analysis of TVR density and proximity to population groups, which can inform future public health and supply reduction policy.

In 2022, all Australian states and territories except Queensland and Victoria had fully implemented various types of tobacco retailer licensing schemes. Queensland will introduce a tobacco retailer licensing scheme in 2024 [11]; understanding the current spatial distribution of tobacco retail outlets in Queensland will provide crucial data for this policy and inform future regulation in line with the National Tobacco Strategy (2022–2023) [12]. In the absence of a Queensland retailer licensing scheme in 2022, geolocation data could be readily obtained from public datasets, such as Google Maps. Therefore, the first study of our research programme aimed to develop and test a prototype system to prospectively collect monthly specialty tobacco/vape retailer location data to estimate the total number of tobacconists and vape stores in Queensland and observe trends in growth over a 12‐month period (2022–2023). If successful, this system will be a research tool that can be used across a variety of retail environments relevant to public health outcomes to efficiently and independently: complement existing retailer licensing databases in tracking longitudinal trends of retail environments to guide and observe the impact of policy; assist with government monitoring of compliance in regard to retailer licensing (i.e., if they currently hold a licence) and supply and advertising laws (e.g., via Google reviews, and Google Streetview storefront and business listing images); and provide baseline and longitudinal data for jurisdictions without a retailer licensing scheme.

## 2 METHODS

This study was approved by the CSIRO Health and Medical Human Research Ethics Committee (Approval Number 074/22).

### 2.1 Search strategy

The ‘Text Search’ access point provided by Google Map's commercial application programming interface (API) was used to obtain the data. A Text Search returns information about a set of places based on a string query, with a specific search location given by a central latitude and longitude coordinate. For this search, two string queries were used, “tobacco shop” and “vape shop” Each API call is limited to 20 business listings within a 50 km radius. The returned data fields are shown in Table S1.

Queensland is the world's sixth‐largest sub‐national entity and larger than all but 16 countries (total area 1.853 million km^2^). Given this vast area, the Google Maps search points (unique latitude and longitude coordinates) were selected from the open‐source ‘Australian Postcode Database’ to maximise coverage while minimising search overlap. Geographic and electoral metadata for each region were obtained. Regions that are unpopulated, typically due to geographical features, were excluded. The number of search points was 3481 and these remained unchanged throughout the study (Figure S1).

### 2.2 Data collection, storage and confirmation

A search was run every month from July 2022 (baseline) to June 2023 on or around the 15th of the month. The search regions and data obtained from Google Maps were placed in a PostgreSQL database. Results from Google Maps were displayed in a web front end following the monthly search and denoted as “New” under the ‘Action’ field (Figure S2).

By means of validation, two team members (HB and DI) independently manually checked and categorised each ‘New’ entry by viewing the Google Maps listing and completing additional business searches if required. Retailers were confirmed as a specialty TVR by considering store name, available storefront/in‐store images (i.e., Google Streetview images or those attached to the Google Maps listing), Google reviews or store directory listing if within shopping centres. Confirmed online specialty TVRs with a physical commercial/residential address in Queensland were also included. Team members categorised retailers by type of store (tobacco only, vape only, and tobacco and vape) and denoted if other items were sold (e.g., gifts, food/drinks). If it was unclear if the store sold both tobacco and vapes, the most likely single option was chosen. If a store was not able to be confirmed using the aforementioned criteria it was categorised as ‘Investigate’, and if the store was not relevant it was categorised as ‘Remove’. Potential duplicate listings were automatically flagged by the database if stores were within a specified radius of each other and then manually checked by comparing addresses. Stores that were not specialty TVRs but likely sold tobacco products (i.e., supermarkets, liquor stores, convenience stores) were categorised as ‘Exclude for now’ and removed for the purposes of this exploratory study. Confirmed specialty TVRs that were not operational (i.e., temporarily or permanently closed) were flagged using the ‘Notes’ field, and stores which were temporarily closed were re‐checked for changes in operational status in subsequent months. Search results without an active Google Maps listing were considered ‘Missing’. Consolidated monthly data was exported into an excel spreadsheet to distribute to the wider team for verification and analysis.

### 2.3 Data analysis

Descriptive statistics of retailer confirmation status were calculated for each month to track growth and the proportion of monthly specialty TVR search results confirmed operational.

## 3 RESULTS

### 3.1 Total TVRs confirmed, operational and monthly changes

**FIGURE 1:**
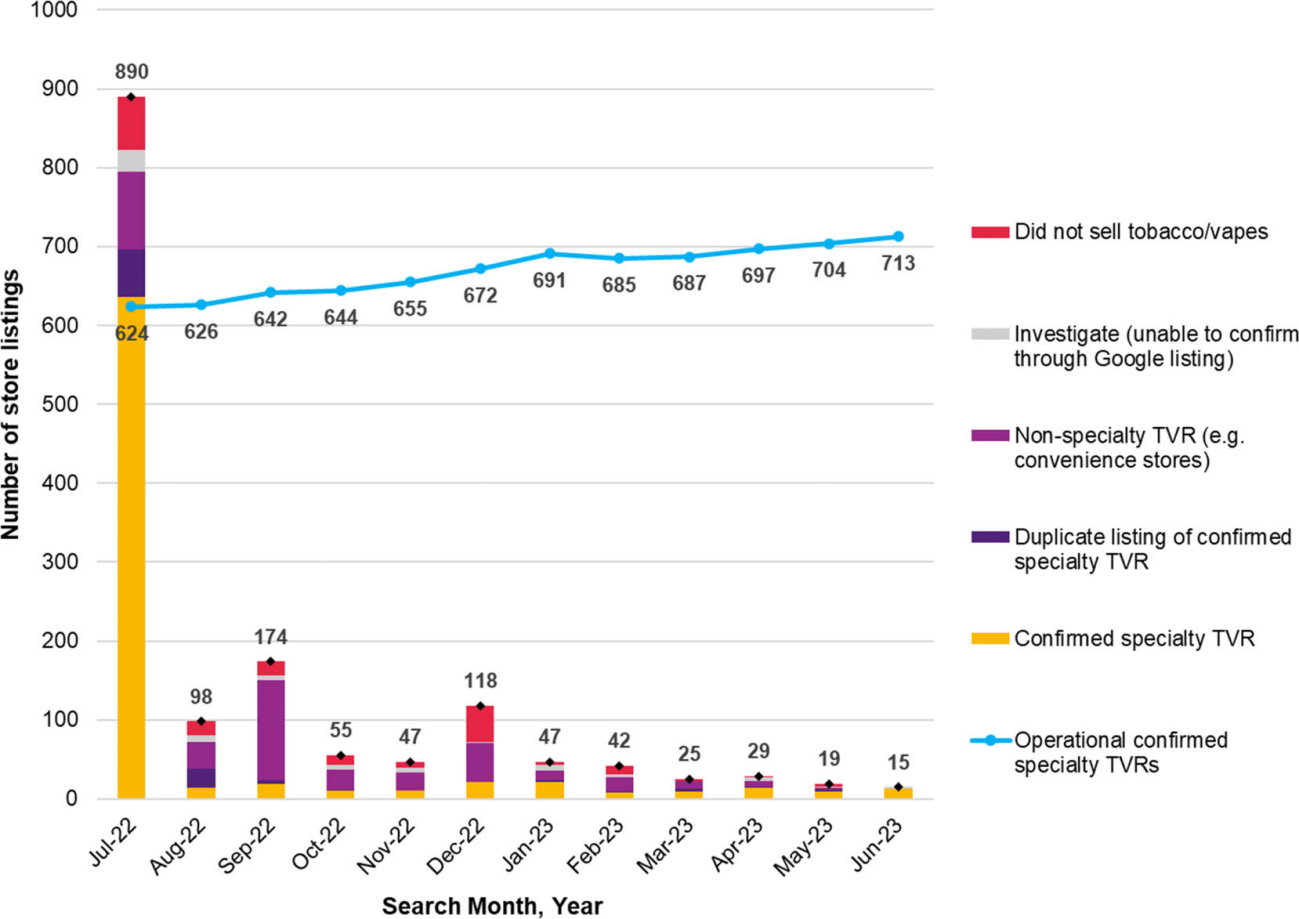
Monthly changes in Google application programming interface search results by confirmation status and in operational tobacconist and vape stores in Queensland between July 2022 and June 2023. TVR, tobacconist and/or vape retailer.

From July 2022 to June 2023, confirmed operational specialty TVRs across Queensland trended upwards, increasing by 14.3% from 624 to 713, a mean of 7.4 new stores per month (Figure 1). The majority of results requiring manual confirmation were found in the first month (*n* = 890) and declined in the following months, with the exception of September and December 2022.

Table 1 shows the total number of stores found per month and the monthly cumulative total of operational TVRs and by store type. Of the total stores collected in the initial search (*n* = 890), almost three quarters (*n* = 636, 71.5%) were manually confirmed as TVRs of which 624 were operational (Table 1). The proportion of confirmed new stores in subsequent monthly search results decreased below 25% between August and December 2022, but generally increased between January and June 2023. The majority of TVRs were categorised as selling ‘tobacco only’ and the proportion of these stores remained relatively consistent over 12‐months, whereas the proportion of ‘tobacco and vape’ stores increased from 24.7% to 30.6% and ‘vape only’ stores decreased from 16.2% to 13.0% (Table 1).

**TABLE 1:** Monthly Google application programming interface search results confirmed as operational TVRs by store type.

Month	Monthly total *N* (%) operational TVRs by store type			
	Vape	Tobacco	Tobacco and vape	
July 2022	101 (16.2)	369 (59.1)	154 (24.7)
August 2022	101 (16.1)	367 (58.6)	158 (25.2)
September 2022	99 (15.4)	378 (58.9)	165 (25.7)
October 2022	97 (15.1)	380 (59.0)	167 (25.9)
November 2022	100 (15.3)	383 (58.5)	172 (26.3)
December 2022	100 (14.9)	388 (57.7)	184 (27.4)
January 2023	102 (14.8)	396 (57.3)	193 (27.9)
February 2023	97 (14.2)	395 (57.7)	193 (28.2)
March 2023	94 (13.7)	397 (57.8)	196 (28.5)
April 2023	94 (13.5)	400 (57.4)	203 (29.1)
May 2023	94 (13.4)	397 (56.4)	213 (30.3)
June 2023	93 (13.0)	402 (56.4)	218 (30.6)

*Note*: Operational: stores that were open for business at the time of search.

Abbreviation: TVR, tobacconist and/or vape retailer.

## 4 DISCUSSION

This study sought to develop and trial a prototype system to collect and consolidate prospective geolocation data for specialty TVRs in Queensland, Australia. Over the 12‐month period, we have demonstrated that the system can be deployed as required for tracking the number and locations of TVRs across Queensland. Furthermore, as the database grew the number of false positive results decreased as evidenced by the monthly increase in the proportion of confirmed new TVRs. We also observed growth in ‘tobacco only’ and ‘tobacco and vape’ stores, and a decline in ‘vape only’ stores. While we cannot make any strong assumptions given the limitations of the data, one hypothesis could be that retailers pivoted in supply in anticipation of, or response to, the Australian Government reforms to regulation of vaping products announced in May 2023 [13], as well as the Queensland Government inquiry into reducing rates of e‐cigarette use announced in March 2023 [14].

This is the first Australian study to attempt to track the growth of the specialty TVR environment on a state‐wide level without a retailer licensing scheme. Previous studies from Western Australia [15, 16], New South Wales [17, 18], Tasmania [19] and South Australia [20] have used existing licensing databases to examine associations between retailer locations and sociodemographic/economic factors, health behaviours, and location characteristics. Conversely, studies from Victoria have relied on a local government list of retailers supplemented by internet searches and observational audits as a means of confirmation [21, 22]. A licensing register allows for more comprehensive, accurate, and efficient evaluations of the state‐wide tobacco retailer landscape in relation to the population. These robust evaluations are necessary to understand commercial determinants of health at play [23, 24] and inform supply reduction policy, thus benefiting populations most at risk. Due to the exploratory and descriptive nature of our study and specific focus on specialty TVRs rather than all tobacco product outlets, we are unable to compare trends observed in other Australian states. As such, the next phase of our research will seek to use this system and future results to assess relationships between specialty TVR density and proximity and population/location characteristics (e.g., sociodemographic, remoteness and youth‐based services) across Queensland prior to the implementation of a licensing register.

During this study, the Queensland government announced plans for a smoking products licensing scheme to be implemented in 2024 [11], and the Australian federal government announced a ban on the sale of disposable vapes from January 1, 2024 [25] and a prescription‐only access model for therapeutic vaping devices via pharmacies, which should ultimately force the closure of vape retailers. Our future search results may provide baseline and periodical geolocation data for the Queensland Government to cross‐reference with for licensing and legislation compliance. Publicly available information included within Google Maps listings, which were used during our manual verification of stores, can also assist with monitoring of compliance with product supply and advertising laws. For example, Google reviews may refer to the sale of banned/illicit products, and storefront and in‐store images may show illegal advertisements or displays of products. As such, ongoing searches will complement government databases to allow for robust longitudinal monitoring and evaluation of the impact both aforementioned, as well as existing, state and federal initiatives.

The longitudinal automated search, with robust quality control through manual confirmation, is a key strength of the system and an improvement over the cross‐sectional observational audit previously employed in similar Australian studies [7, 21, 22, 26]. The ability to easily monitor trends in ‘real time’, perhaps in response to policy and other factors, is also a distinct advantage. Furthermore, the system can also be used to gather location data on other types of retailers and services associated with negative (e.g., liquor stores, fast‐food outlets, gambling venues) and positive (e.g., fresh‐food stores, private health services) health outcomes. However, there are limitations to our system and study. Firstly, we were limited in time and resources to conduct monthly field verification on a state‐wide level. The accuracy of the Google Maps data may be limited as business entries can be created by store owners and reviewers/customers, but we attempted to mitigate this by the removal of duplicates and manual checking of store metadata, reviews, and photos. While our search did collect some online retailers based in Queensland (e.g., linked to a commercial/residential address), the vast majority of results were physical store locations and likely did not capture all retailers that extend beyond traditional ‘brick‐and‐mortar’ stores. Although our manual verification process can be seen as a strength, reliance on this method is also a limitation due to the time required. However, we will improve and further automate this process by training an AI algorithm to detect keywords in business listing information or reviews, or objects in photos attached to google listings to reduce the need for manual verification.

## 5 CONCLUSION

The prototype system developed in this study effectively identified and tracked the growth of tobacconists and vape stores in Queensland over 12 months. Future searches using the system will provide baseline data to assist compliance during the introduction of the Queensland smoking product licensing scheme and longitudinal data to supplement evaluations of the state and federal supply policies. Future research will use this system to collect state‐wide location data to investigate associations between measures of TVR density/proximity and population and location characteristics.

## AUTHOR CONTRIBUTIONS

Each author certifies that their contribution to this study meets the standards of the International Committee of Medical Journal Editors.

## FUNDING INFORMATION

This project was supported by funding from the Prevention Strategy Branch, Queensland Health.

## CONFLICT OF INTEREST STATEMENT

The authors have no further conflicts of interest to declare, and there are no constraints on publishing.

## Supporting information


**TABLE S1.** Description of fields returned from the Text Search application programming interface call.
**FIGURE S1.** Map of Queensland showing locations searched by Google Maps application programming interface (Locations correspond to registered localities in Queensland).
**FIGURE S2.** Screenshot of web page user‐interface to allow for manual confirmation, notes and additional metadata.

## Data Availability

The data that support the findings of this study are available from the corresponding author upon reasonable request.
