# Supplementary material for: Estimating the number and growth of tobacconists and vape stores in Queensland in the absence of a retailer licensing database
Source: Drug Alcohol Rev. 2025 Mar 3;44(4):1108–13. doi: 10.1111/dar.14038 (PMC12117295; doi:10.1111/dar.14038)
Supplement: Supplementary file 1 — TABLE S1. Description of fields returned from the Text Search application programming interface call. FIGURE S1. Map of Queensland showing locations searched by Google Maps application programming interface (Locations correspond to registered localities in Queensland). FIGURE S2. Screenshot of web page user‐interface to allow for manual confirmation, notes and additional metadata. [file DAR-44-1108-s001.docx]

**Supplementary Files –** *Estimating the number and growth of tobacconists and vape stores in Queensland in the absence of a retailer licensing database*

**Supplementary Table 1**: Description of fields returned from the Text Search API call

| **Field** | **Description** |
| --- | --- |
| Place ID | A place ID is a textual identifier that uniquely identifies a place in the Google Map’s database. |
| Name | The name of the business. |
| Address | The address of the business. |
| Geometry | Latitude & longitude coordinate of the business. |
| Business Status | The status of the business e.g. ‘operational’, ‘temporarily closed’, and ‘permanently closed’ |
| Opening Hours | Operating times of the business and whether the business is open at the time of search. |
| Photos | URL links to uploaded photos of the business. |
| Rating | The rating score of the business. |
| User Ratings Total | The total number of ratings of the business. |

**Supplementary Figure 1:** Map of Queensland showing locations searched by Google Maps API (Locations correspond to registered localities in Queensland)

**Supplementary Figure 2:** Screenshot of web page user-interface to allow for manual confirmation, notes and additional metadata


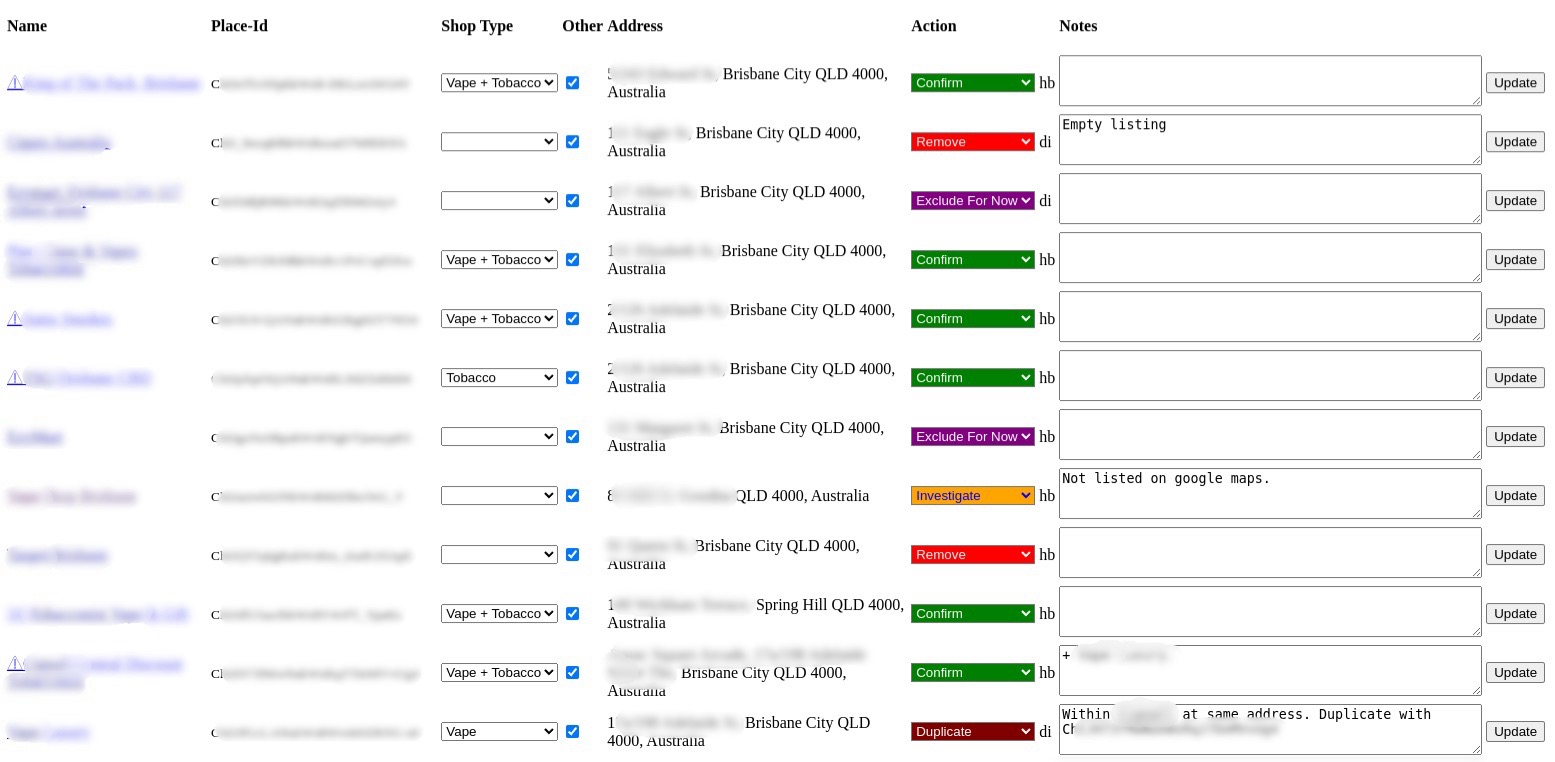


**Name, Place-Id, and Address have been removed for privacy reasons*
